# The Normal Hip Score Following Hip Fracture: Preliminary Validation of a Single-Item Patient-Reported Outcome Measure

**DOI:** 10.3390/jcm15208078

**Published:** 2026-10-17

**Authors:** Brian Z. H. Lim, Gillian C. Leitch, Thomas R. Williamson, Andrew D. Duckworth, Nick D. Clement

**Affiliations:** 1 Edinburgh Orthopaedics, Royal Infirmary of Edinburgh, Little France Crescent, Edinburgh EH16 4SA, UK; 2 Usher Institute, School of Population Health Sciences, University of Edinburgh, Edinburgh EH16 4UX, UK

**Keywords:** Normal Hip Score, EQ-5D, Oxford hip score, hip, fracture, outcome

## Abstract

**Background:** Patient-reported outcome measures (PROMs) are important after hip fracture but can impose respondent burden. A simple global measure may provide complementary information about a patient’s overall perception of hip normality. The Normal Hip Score (NHS) is a single-item 0–100 measure of perceived hip normality. This study evaluated preliminary evidence of construct validity for the NHS following hip fracture surgery. **Methods:** This was a prospective observational study with cross-sectional validation analyses based on a single postoperative PROM assessment. Paired NHS–OHS and NHS–EQ-5D-5L data were available for 80 participants. The mean age was 79.3 years (SD 10.8), and 46 (57.5%) of these participants were female. Assessment occurred at a mean of 3.47 months (SD 0.75; range 2–7 months) following fracture surgery. The NHS was assessed in relation to the OHS and EQ-5D-5L index and dimensions. Convergent validity was assessed using Pearson and Spearman correlations, with exploratory analyses of individual OHS items and EQ-5D-5L dimensions. Known-groups validity was examined across OHS quartiles, and multivariable linear regression was performed. **Results:** The mean NHS was 60.4 (SD 25.4). NHS demonstrated a moderate correlation with OHS (Pearson r = 0.657, 95% CI 0.511–0.766; *p* < 0.001) and with the EQ-5D-5L index (r = 0.541, 95% CI 0.365–0.680; *p* < 0.001). All 12 OHS items and all five EQ-5D-5L dimensions were significantly associated with NHS. The strongest OHS item associations were pain when rising from a chair, pain, work/housework interference, limping, and washing/bathing. Mobility showed the strongest EQ-5D-5L dimension association. NHSs increased across OHS quartiles (Kruskal–Wallis H = 35.77; *p* < 0.001). In multivariable analysis, OHS remained associated with NHS (β = 1.54, 95% CI 0.80–2.28; *p* < 0.001). **Conclusions:** The NHS demonstrated preliminary evidence of cross-sectional construct and known-groups validity following hip fracture surgery. The findings support the NHS as a potentially useful complementary global measure of perceived hip normality, but do not establish test–retest reliability, responsiveness, clinically meaningful change, or suitability for standalone clinical use.

## 1. Introduction

Hip fractures are a major cause of mortality, disability, and loss of independence in older adults [1,2]. Assessment of recovery from hip fracture has increasingly incorporated patient-reported outcome measures (PROMs), which provide information about health status and functional recovery [3]. Although a number of PROMs are used following hip fracture, few have been specifically developed or comprehensively validated in this patient population [4]. Hip fracture patients are typically older and frailer than those undergoing elective hip surgery and have higher levels of multimorbidity, immobility and cognitive impairment [5,6]. These characteristics may influence both the ability to reliably complete the scores, as well as the capacity of conventional PROMs to capture outcomes that are most relevant to this patient population.

The EQ-5D is an established measure of health-related quality of life (HRQoL) and is commonly used to assess outcomes following hip fracture [7]. It is widely used in clinical studies, national audits, and registries evaluating hip-fracture outcomes. Nevertheless, it is a generic measure of health status rather than a hip-specific PROM [8]. Recovery following hip fracture involves changes in hip pain, mobility, function, gait, and the ability to undertake activities that depend specifically on hip function. A generic HRQoL measure may therefore not fully capture the patient’s perception of hip recovery. The Oxford Hip Score (OHS) is a validated hip-specific PROM originally developed to assess pain and functional limitations in patients undergoing total hip arthroplasty, but can provide complementary information to the EQ-5D when evaluating recovery following hip fracture [7,9]. However, the 12-item format imposes a greater questionnaire burden than a single-item measure, which may be particularly relevant in the hip fracture population. Guidance from the PROM Working Group of the International Society of Arthroplasty Registries emphasises selecting PROMs that retain essential information while minimising respondent burden; this guidance was developed for arthroplasty registries and is therefore relevant here as general PROM guidance rather than as hip-fracture-specific validation evidence [10].

**Figure 1:**
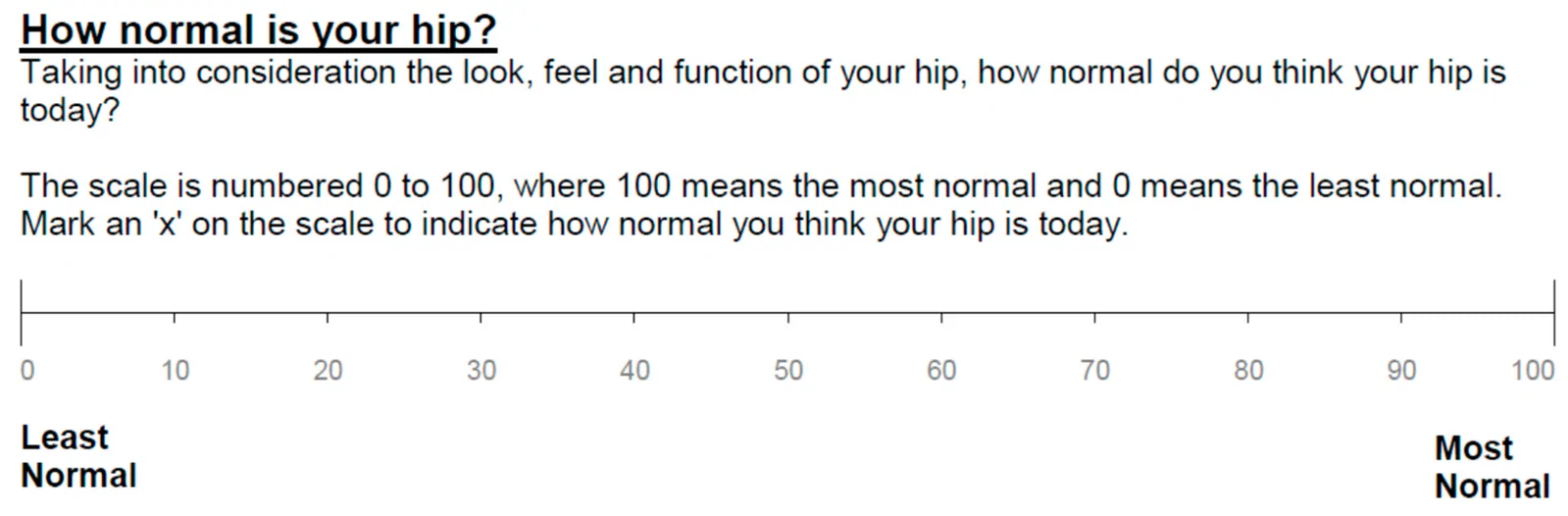
Normal Hip Score (NHS) questionnaire. The NHS is a single-item patient-reported measure of perceived hip normality. Patients are asked: “How normal is your hip? Taking into consideration the look, feel and function of your hip, how normal do you think your hip is today?” The response scale ranges from 0 (least normal) to 100 (most normal).

Therefore, there is a need for a simple and patient-centred measure that captures the overall perception of how normal the hip feels, looks, and functions following hip fracture surgery, while minimising respondent burden. The Single Assessment Numerical Evaluation (SANE) is a related global, single-item measure in which patients rate the current status of an affected body region on a 0–100 scale, with 100 representing normal [11]. In a hip-specific study, SANE showed moderate correlations with established hip-specific PROMs [12]. However, that study involved a predominantly younger hip-preservation/elective population rather than older patients recovering from hip fracture. The NHS uses a similar 0–100 single-item format but asks specifically, “How normal is your hip?”, taking into consideration the look, feel and function of the hip (Figure 1). Thus, the NHS is conceptually related to, but distinct from, SANE and has not previously been evaluated in a hip-fracture population. This is the first study to evaluate the validity of the NHS.

A related single-item measure is the Subjective Hip Value (SHV) [13,14], which was developed analogous to the Subjective Shoulder Value [15]. The SHV asks patients to rate the overall “value” of their hip as a percentage of a completely normal hip, with an alternative monetary formulation available when patients have difficulty with the question [13]. The NHS is conceptually related to and anchored to a completely normal hip. However, the NHS was designed specifically to assess perceived hip normality rather than the subjective value assigned to the hip. The NHS question explicitly asks patients to consider the look, feel and function of their hip and to rate how normal it is today. Thus, although both measures provide a global single-item assessment relative to a normal hip, they differ in the construct and wording used to elicit the patient’s judgement. The present study evaluates the NHS specifically in patients recovering from hip fracture, a population distinct from the clinical populations in which the SHV has previously been described [13,14].

The aim of this study was to evaluate the preliminary construct validity of the NHS following hip fracture surgery. Specifically, associations with the OHS and EQ-5D-5L index were explored, together with exploratory relationships between NHS and individual OHS items and EQ-5D-5L dimensions. We also assessed known groups’ validity according to OHS performance and examined whether NHSs were associated with age, sex, or time from fracture to assessment.

## 2. Methods

### 2.1. Study Design and Setting

This was a prospective observational cohort study conducted in adult patients treated surgically for hip fracture at a single tertiary orthopaedic centre. The present validation analyses were cross-sectional, based on PROMs collected at a single postoperative assessment. Ethical approval was obtained for PROM data collection and analysis under the Edinburgh Orthopaedic Research Database (Scotland B REC, number 25/SS/0049-IRAS-353690).

### 2.2. Participants

Patients undergoing operative management for an acute hip fracture between July and October 2025 were considered for inclusion. Eligibility criteria were age ≥ 50 years, a radiologically confirmed hip fracture (femoral neck, intertrochanteric, or subtrochanteric), and the ability to complete PROMs independently or with consistent proxy assistance, as per all standardised outcome PROMs by this department. Patients with pathological fractures or cognitive impairment precluding meaningful PROM completion were excluded. PROMs were completed by patients postoperatively via a postal questionnaire. Cognitive eligibility was assessed prior to sending out the questionnaire, and patients who were deemed frail (e.g., nursing home residents) or had cognitive impairment were not included in the eligible cohort. Some patients did return the questionnaire not completed, stating that they were not able to complete it, often via a proxy, due to frailty and/or cognitive decline.

### 2.3. Data Collection and Patient-Reported Outcome Measures

Baseline demographic and clinical data, including age and sex, were recorded. PROMs were collected at a single postoperative assessment, with the time from surgery to assessment recorded. The NHS is a single-item patient-reported measure of perceived hip normality (Figure 1), developed with patient involvement incorporated into the process in accordance with COSMIN recommendations for the development and evaluation of health status measurement instruments [16]. Patients contributed to the development and refinement of the question wording and response format to ensure that the measure was understandable, relevant, and reflected patients’ perceptions of hip recovery. The NHS was developed with patient involvement before the present validation study. Ten patients undergoing total hip arthroplasty contributed to the development/refinement of the question. Feedback was obtained through both individual interviews and group discussions and was used to refine accordingly. Particular attention was paid to whether patients understood ‘normal’ as their own ideal healthy hip. No comparison was made in relation to the contralateral hip, as the patient group felt that some individuals may have hip pathology and therefore may not be “normal”. The final wording was selected following this process and was: ‘How normal is your hip? Taking into consideration the look, feel and function of your hip, how normal do you think your hip is today?’ Patients marked a position on a 0 to 100 numerical rating scale, where 0 represented the least normal hip, and 100 represented the most normal hip.

The OHS is a validated 12-item hip-specific patient-reported outcome measure assessing pain and functional limitation [9]. Each item is scored from 0 to 4, generating a total score from 0 to 48, with higher scores representing better hip-related health [17]. The EQ-5D-5L is a generic measure of HRQoL comprising five dimensions: mobility, self-care, usual activities, pain/discomfort, and anxiety/depression [18]. The EQ-5D-5L index has also been explicitly linked to the UK EQ-5D-5L value set (Rowen et al. [19]), based on EQ-VT v2.1, with higher values indicating better HRQoL.

### 2.4. Statistical Analysis

Descriptive and inferential statistical methods were used. All statistical analyses were performed using IBM SPSS Statistics for Windows, version 17.0 (SPSS Inc., Chicago, IL, USA). Continuous variables were summarised using means and standard deviations and medians and ranges, as appropriate, while categorical variables were summarised as frequencies and percentages. Construct validity was assessed using Pearson and Spearman correlation coefficients to examine associations between NHS and OHS total scores and EQ-5D-5L index scores. A priori, correlations of ≥0.40 were considered to represent acceptable evidence of convergent validity; this threshold was specified before examination of the observed correlations. Ninety-five percent confidence intervals (95% CI) were calculated for the principal Pearson correlation coefficients. Exploratory item-level analyses examined associations between the NHS and each of the 12 OHS items and each of the five EQ-5D-5L dimensions. Pearson correlation coefficients were used to assess linear associations, and Spearman rank correlation coefficients were used as a non-parametric assessment of monotonic associations.

Associations between NHS and age and time from fracture to assessment were assessed using correlation analysis. Differences in NHSs according to sex were examined using an independent-samples *t*-test. Known-groups validity was assessed by dividing participants into quartiles according to OHS total score and comparing NHSs between groups using the Kruskal–Wallis test; because these groups were derived from the same OHS measure used for the primary convergent-validity analysis, this was considered supportive/exploratory rather than independent validation. A multivariable linear regression model was constructed with NHS as the dependent variable and OHS total score, EQ-5D-5L index score, age, sex, and time from fracture to assessment as independent variables. Regression coefficients (β), 95% confidence intervals, *p*-values, R^2^, and adjusted R^2^ were reported.

All statistical tests were two-sided, and a *p*-value < 0.05 was considered statistically significant. Given the exploratory nature of the item-level analyses, these findings were interpreted primarily according to the magnitude and direction of associations rather than statistical significance alone.

### 2.5. Sample Size

For the primary convergent-validity analyses, we prespecified that a correlation of approximately 0.40 or greater would represent a potentially meaningful association, reflecting a moderate relationship between the NHS and established PROMs while recognising that the NHS was intended to capture a related but non-identical construct. This threshold was used as a descriptive criterion rather than as a definitive test of validity. We hypothesised that NHS would demonstrate a positive association with OHS and the EQ-5D-5L index, and negative associations with EQ-5D-5L impairment dimensions. Because the NHS was intended to represent global hip normality rather than a specific symptom domain, no formal ranking of individual OHS items was prespecified.

A minimum target sample of 80 participants was specified to provide an adequate sample for the preliminary validation analysis. Assuming an anticipated correlation coefficient of 0.40 between the NHS and an established patient-reported outcome measure, a sample of approximately 80 participants provides approximately 90% power to detect such an association at a two-sided alpha level of 0.05 using Fisher’s z transformation.

## 3. Results

### 3.1. Study Population

**Figure 2:**
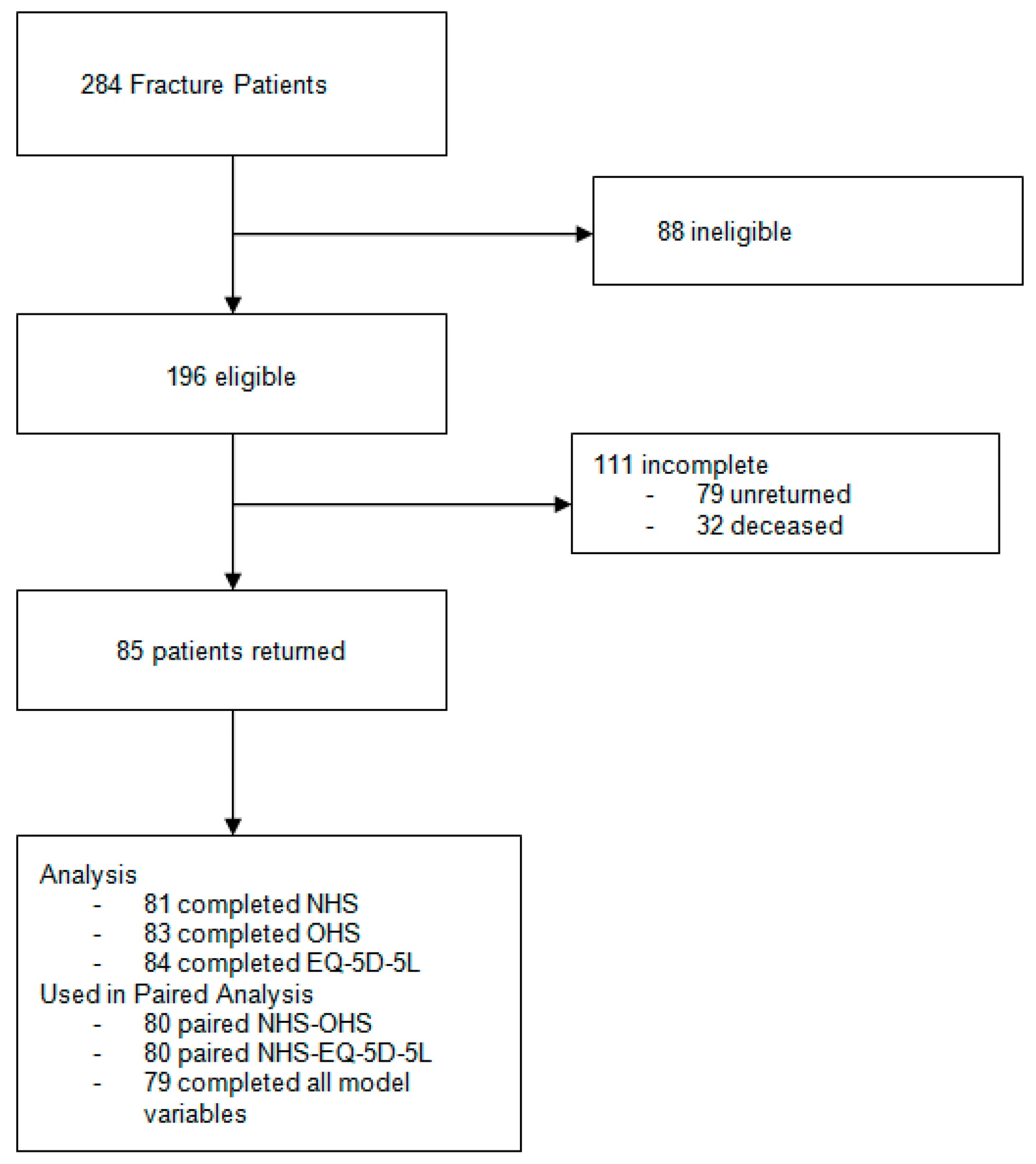
Participant flow diagram from identification to subgroup analysis.

Of 196 eligible patients, 85 returned questionnaires (43.4%), at a mean time from fracture of 3.47 months (SD 0.75). Thirty-two (28.8% of 111 unreturned questionnaires) patients died before questionnaire completion, while 79 (71.2% of 111 unreturned questionnaires) did not return the questionnaire; only 11 patients stated they were not able to do so because they were too frail (often reported by a proxy). There were no completed questionnaires that indicated completion by proxy. Where baseline data were available, characteristics of responders and non-responders were compared, and the only difference was in age, with non-responders being older (79.3 vs. 84.7 years, *p* < 0.001). Participants with incomplete data were excluded from individual analyses as appropriate: 81 participants had an NHS, 83 had an OHS, and 84 had an EQ-5D-5L index score. Paired NHS and OHS data were available for 80 participants, while 80 had paired NHS and EQ-5D-5L data. The multivariable regression analysis included 79 participants with complete data for all model variables (Figure 2).

### 3.2. Population Analysis

There was no single analysis population because the availability of individual PROMs differed. The mean age was 79.3 years (SD 10.8), with 46 (57.5%) participants being female. Most lived independently, with only two (2.5%) living in a care home. The most frequently performed operation was hemiarthroplasty (n = 38, 47.5%), followed by dynamic hip screw fixation (n = 20, 25.0%). Total hip arthroplasty accounted for eight cases (10.0%), cannulated screw fixation was performed in five cases (6.2%), and cephalomedullary nailing was performed in nine cases (11.2%). The mean NHS was 60.4 (SD 25.4), with a median score of 60 (range 0 to 100). Among the 81 patients with an NHS, 3 (3.7%) scored 0 and 2 (2.5%) scored 100. Four patients (4.9%) scored ≤ 10, while 15 (18.5%) scored ≥ 90. These findings indicate limited floor effects but some clustering towards the upper end of the scale. The mean OHS was 28.0 (SD 11.2), with a median of 28 (range 0 to 48). The mean EQ-5D-5L index was 0.651 (SD 0.308), with a median of 0.758 (range −0.489 to 1.000).

### 3.3. Convergent Validity with the Oxford Hip Score and EQ-5D-5L

**Figure 3:**
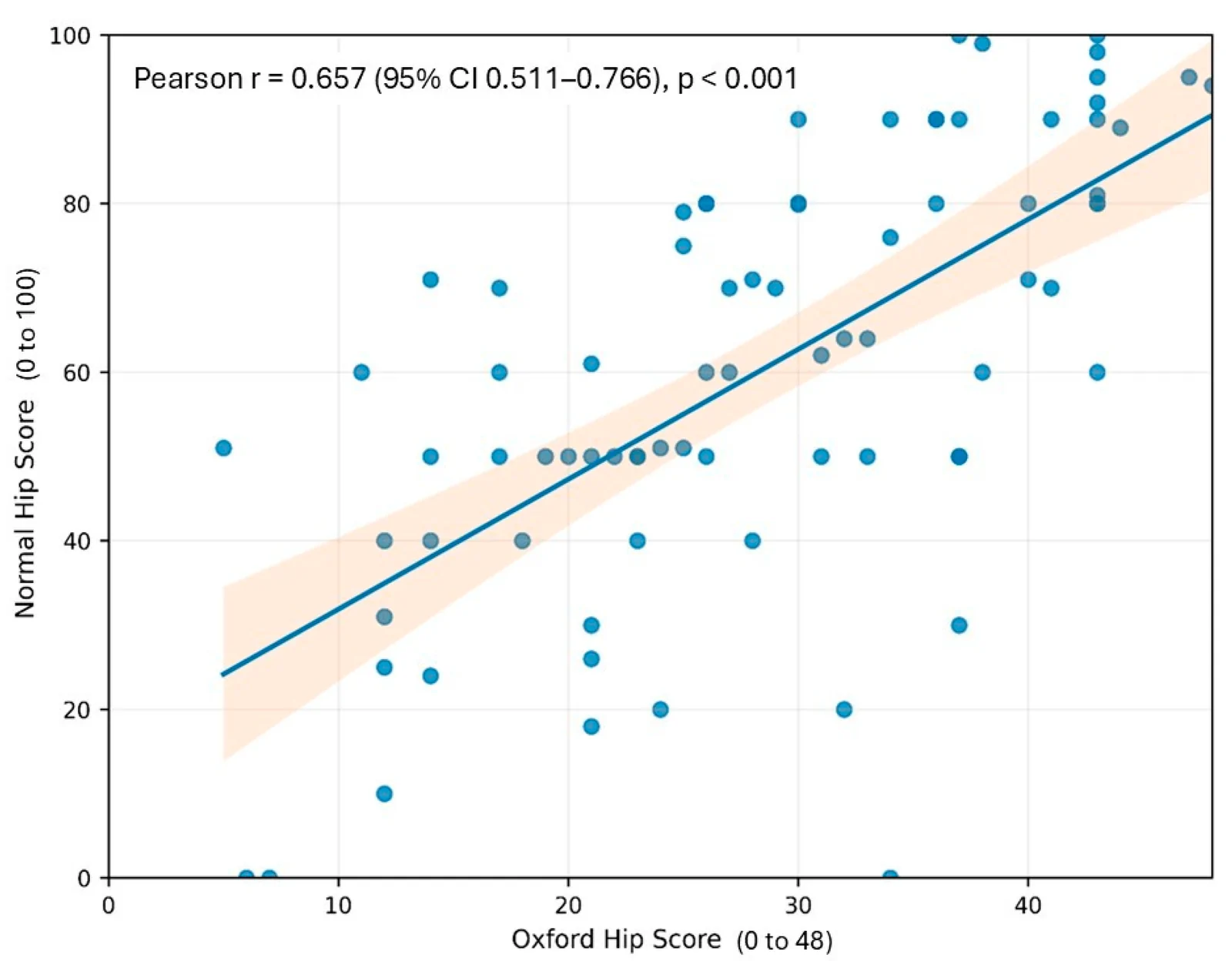
Association between Normal Hip Score and Oxford Hip Score. Scatter plot showing the relationship between NHS and OHS among 80 patients with complete paired data. Higher scores indicate greater perceived hip normality and better hip-related outcomes, respectively. Pearson correlation was r = 0.657 (95% CI 0.511–0.766; *p* < 0.001).

The NHS demonstrated a moderate-to-strong (Pearson’s correlation r = 0.657, 95% CI 0.511–0.766; *p* < 0.001) positive association with the OHS (Figure 3). The estimated relationship was NHS = 16.46 + (1.54 × OHS) (Pearson r = 0.657; R^2^ = 0.432; n = 80). Thus, each one-point increase in OHS corresponded to an estimated 1.54-point increase in NHS. However, as the model explained 43.2% of the variance in NHS, these values should be regarded as population-level estimates rather than interchangeable individual scores.

The NHS demonstrated a moderate positive association with the EQ-5D-5L index. Pearson’s correlation was r = 0.541 (*p* < 0.001), with a similar Spearman correlation (ρ = 0.546; *p* < 0.001). The EQ-5D-5L index explained approximately 29% of the variance in NHS (R^2^ = 0.293).

### 3.4. Association with Individual OHS Items

**Figure 4:**
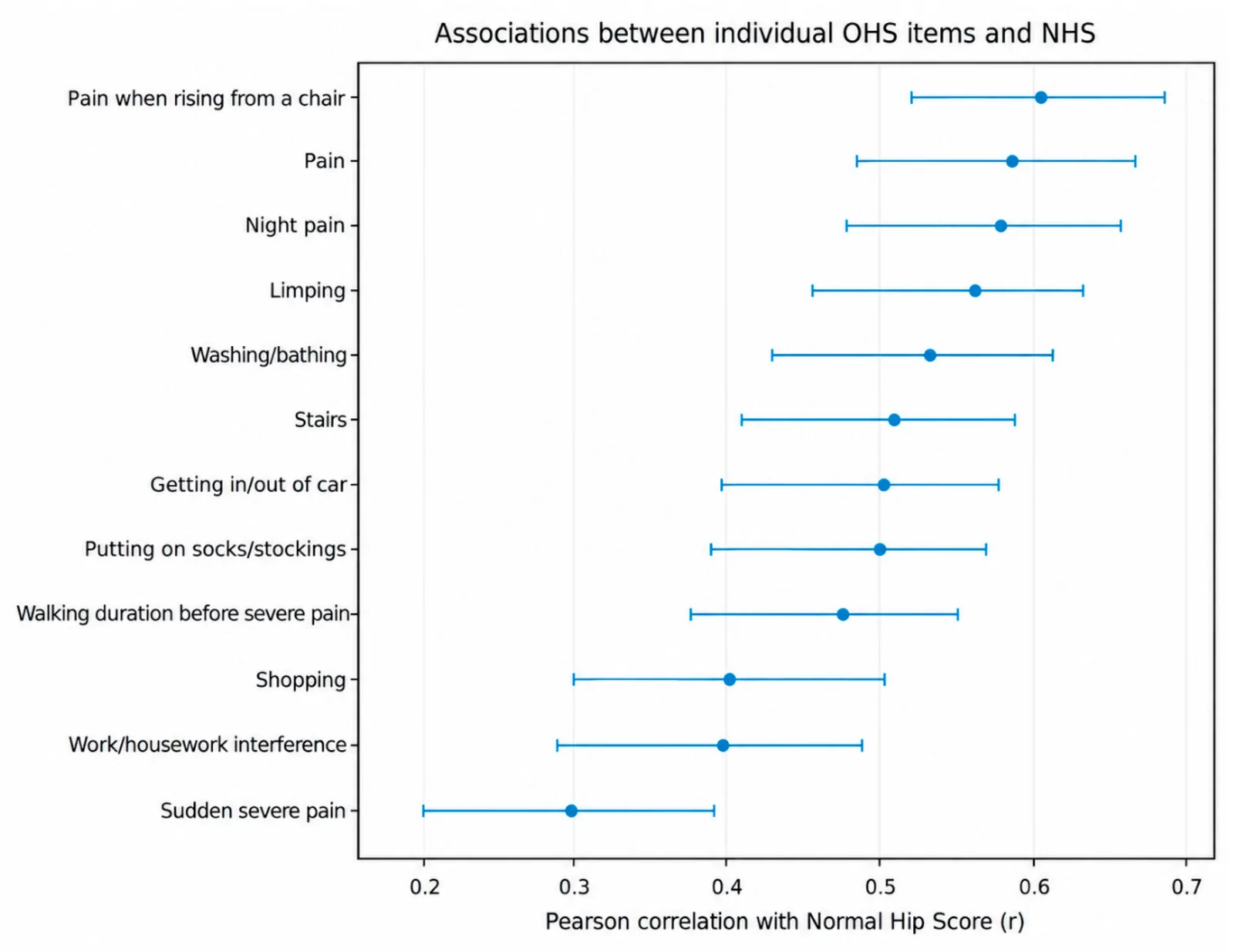
Associations between individual Oxford Hip Score items and Normal Hip Score. Pearson correlation coefficients between each of the 12 OHS items and the NHS in 80 patients with complete item-level data. The strongest associations were observed for pain when rising from a chair, pain, work/housework interference, limping, and washing/bathing; the weakest was for sudden severe pain.

All 12 OHS items were significantly associated with NHS (Table 1, Figure 4). All 12 OHS items were associated with NHS. The strongest correlations were observed for pain when rising from a chair (r = 0.602), pain (r = 0.586), work/housework interference (r = 0.580), limping (r = 0.559), and washing/bathing (r = 0.532). The weakest association was observed for sudden severe pain (r = 0.294).

### 3.5. Association with EQ-5D-5L Dimensions

**Figure 5:**
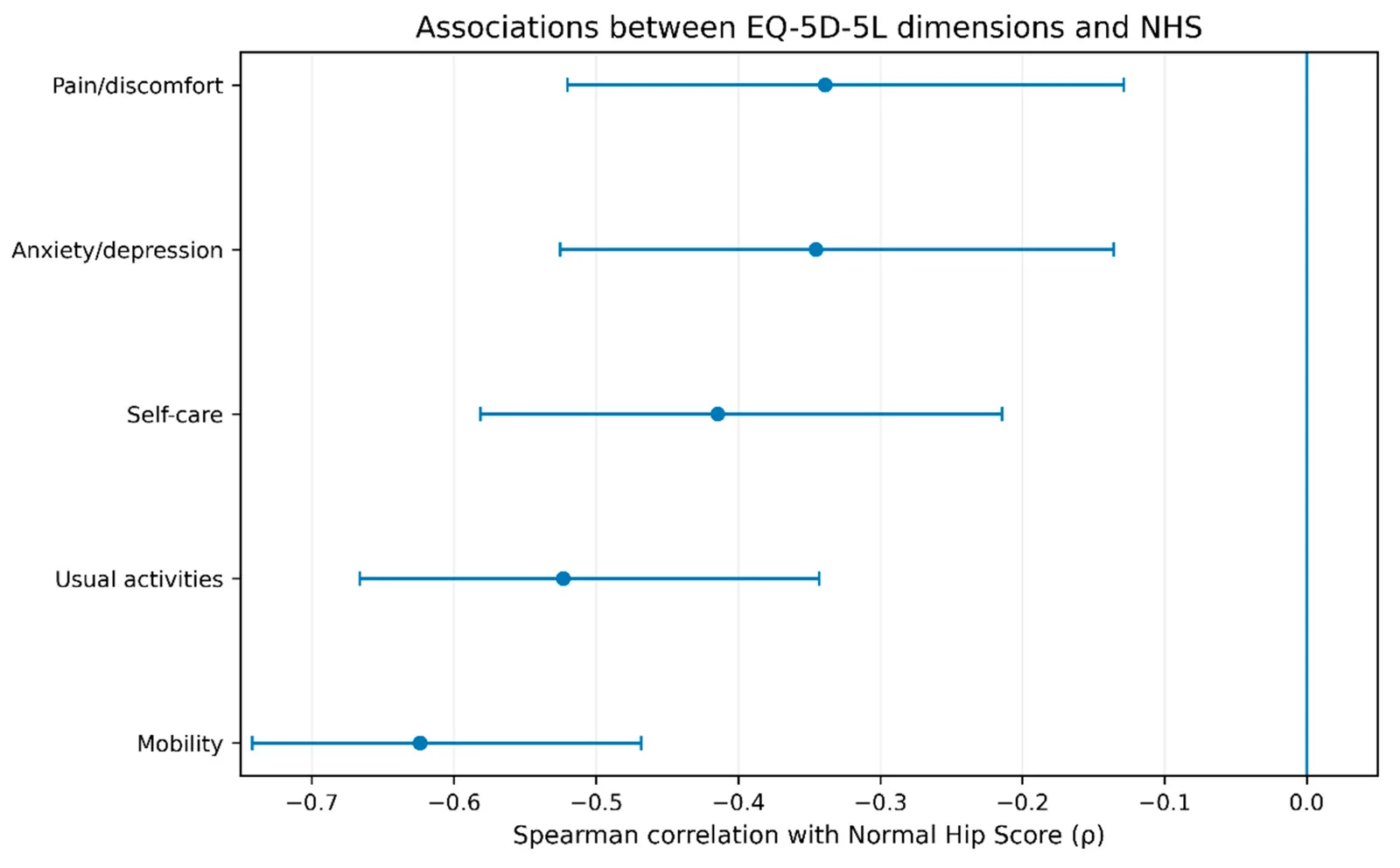
Associations between EQ-5D-5L dimensions and Normal Hip Score. Spearman correlation coefficients between the five EQ-5D-5L dimensions and the NHS in 80 patients with complete paired data. Because higher EQ-5D-5L dimension scores indicate greater problems, correlations are negative. Mobility showed the strongest association with NHS (ρ = −0.624), followed by usual activities (ρ = −0.523).

The NHS was significantly associated with all five EQ-5D-5L dimensions (Table 2, Figure 5). Because higher EQ-5D-5L dimension scores represent greater impairment, correlations were negative. The strongest association was observed with mobility (Spearman ρ = −0.624), followed by usual activities (ρ = −0.523) and self-care (ρ = −0.415). Associations with pain/discomfort (ρ = −0.339) and anxiety/depression (ρ = −0.321) were weaker but remained statistically significant.

### 3.6. Age, Sex and Time Since Surgery

**Figure 6:**
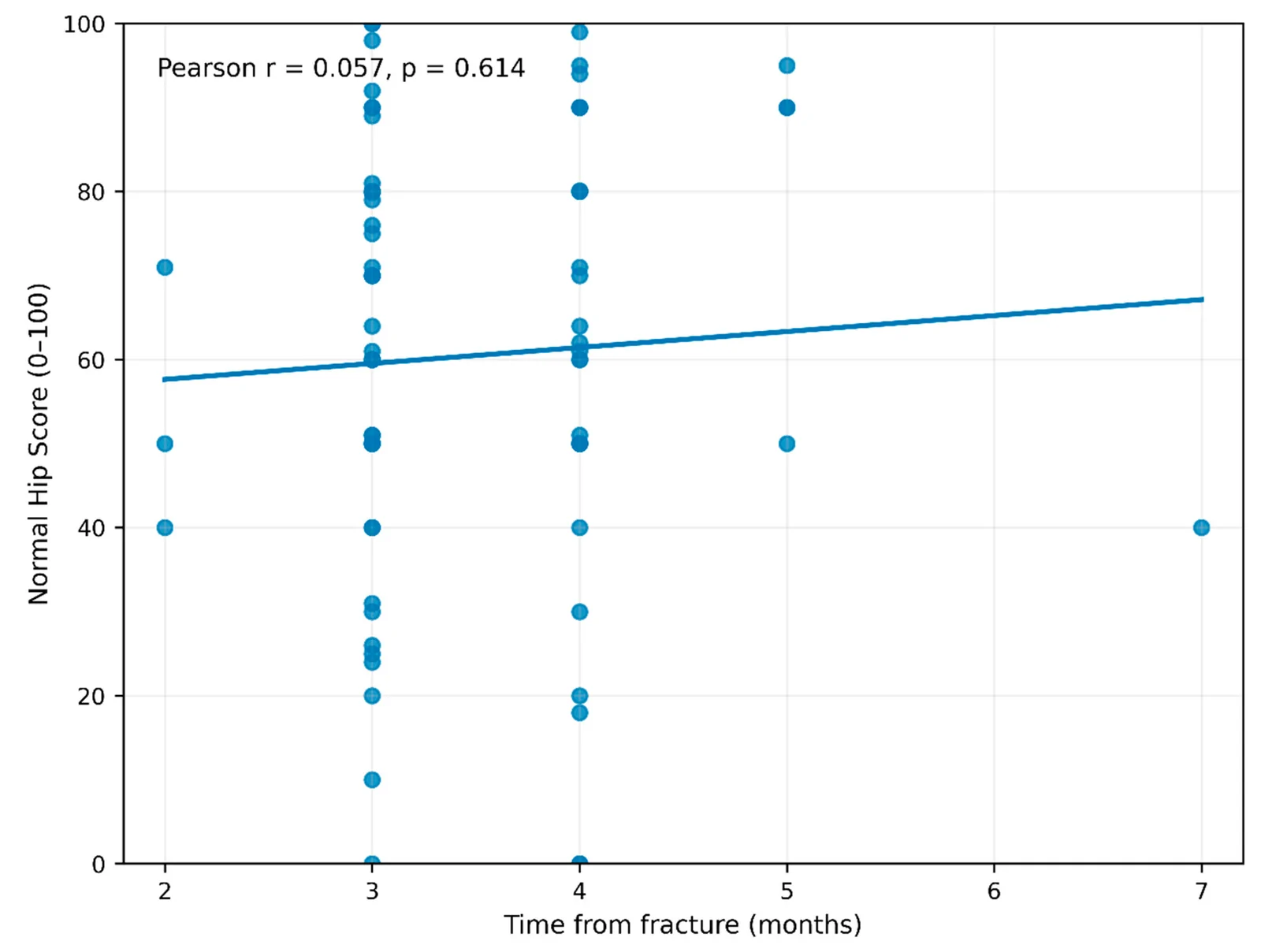
Association between time from fracture and Normal Hip Score. Scatter plot showing the relationship between time from fracture to PROM assessment and NHS among 81 patients with available NHS and timing data. There was no significant association between time from fracture and NHS (Pearson r = 0.057; *p* = 0.614).

There was no statistically significant association between NHS and age (r = −0.113, *p* = 0.314). There was also no significant difference in NHS according to sex. Mean NHS was 57.7 (SD 25.8) among males and 62.4 (SD 25.1) among females (*p* = 0.408). Time from surgery to assessment, within the observed 2-to-7-month period, was not significantly associated with NHS (r = 0.057, *p* = 0.614) (Figure 6).

### 3.7. Multivariable Analysis

A complete-case multivariable linear regression model including OHS, EQ-5D-5L index, age, sex and time since surgery included 79 patients and explained 43.1% of the variance in NHSs (R^2^ = 0.431; adjusted R^2^ = 0.392). OHS remained independently associated with NHS (β = 1.54, 95% CI 0.80–2.28; *p* < 0.001) (Table 3). In contrast, age (β = 0.08, 95% CI −0.36 to 0.53; *p* = 0.720), sex (β = −1.91, 95% CI −11.36 to 7.53; *p* = 0.688), time since surgery (β = 0.35, 95% CI −5.84 to 6.54; *p* = 0.911), and EQ-5D-5L index (β = 1.00, 95% CI −24.74 to 26.73; *p* = 0.939) were not independently associated with NHS.

### 3.8. Known-Groups Analysis According to OHS Quartile

**Figure 7:**
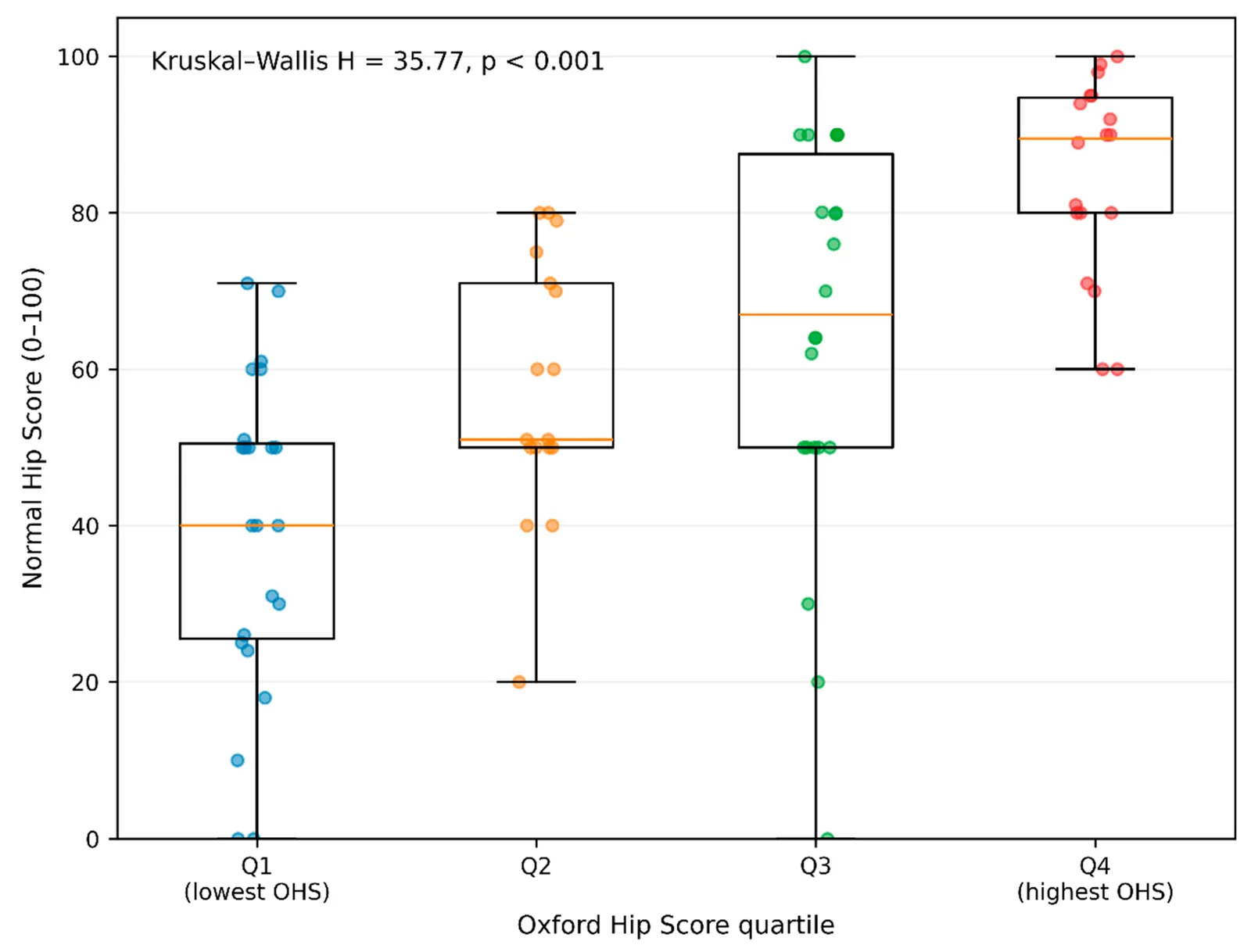
Normal Hip Score according to Oxford Hip Score quartile. Distribution of NHS according to OHS quartiles among 80 participants with paired data. NHSs increased across OHS quartiles (Kruskal–Wallis H = 35.77; *p* < 0.001). Because quartiles were derived from OHS, this analysis is supportive/exploratory rather than an independent validation.

There was a marked gradient in NHS according to OHS quartile (Figure 7). Mean NHS increased from 39.4 in the lowest OHS quartile to 84.7 in the highest quartile, with a highly significant between-group difference (Kruskal–Wallis H = 35.77, *p* < 0.001).

## 4. Discussion

This study provides preliminary evidence supporting the construct validity of the NHS as a patient-reported measure of perceived hip normality following hip fracture. The NHS demonstrated a moderate-to-strong correlation with the OHS and a moderate correlation with the EQ-5D-5L index. All 12 individual OHS items were significantly associated with NHSs, with the strongest relationships observed for standing, pain, night pain, and limping. Among the EQ-5D-5L dimensions, mobility demonstrated the strongest association with NHS, followed by usual activities and self-care. NHSs also demonstrated clear discrimination across OHS quartiles, supporting known-groups validity. In contrast, NHSs were not significantly associated with age, sex, or time from surgery to assessment, supporting generalised use in this population. In multivariable analysis, OHS remained independently associated with NHS, whereas the EQ-5D-5L index did not. Overall, these findings suggest that the NHS captures a meaningful patient-centred perception of recovery that is closely related to hip-specific symptoms and function, while retaining a distinct conceptual role from existing multidimensional PROMs.

The NHS should also be considered in the context of the SANE, an established single-item global PROM using a similar 0 to 100 response format. The psychometric properties of the SANE have been investigated in patients with lower extremity pathologies [20]. The present findings are broadly consistent with this literature, with the NHS demonstrating a moderate correlation with the OHS. However, the populations and constructs differ substantially. The SANE literature has largely involved younger or middle-aged populations with elective hip or sports-related pathology [12,21,22,23]. In a large cohort of patients presenting with hip pain, Bergman et al. [12] reported moderate associations between SANE and several hip-specific PROMs, including iHOT-12 (r = −0.40), Hip Outcome Score (r = 0.57), and HOOS (r = −0.55) [12]. The NHS–OHS correlation observed in the present study (r = 0.657) is therefore broadly comparable in magnitude, although direct comparison is limited by differences in patient population, comparator instruments and study design. Furthermore, the NHS asks specifically how normal the hip is, incorporating look, feel and function, rather than asking patients to “rate your (e.g., injured limb) today”. This concept of a normal joint score has also been explored in upper-limb conditions using the normal hand [24,25] and normal wrist scores [26]. The findings demonstrate that global assessments of joint normality are related to, but not representative of, multidimensional PROMs and may therefore provide complementary information. Nevertheless, the validity of such a score as a standalone measure from which clinical conclusions can be drawn has not yet been established. Thus, the existing SANE and NHS literature provides important precedent for the feasibility, validity and potential utility of a single-item global assessment.

The NHS should also be distinguished from the SHV [13,14], which provides an important precedent for a single-item global assessment of the hip. The SHV asks patients to estimate the overall percentage value of their hip relative to a completely normal hip, with a monetary formulation offered as an alternative when required [13,14]. Both measures therefore use a global judgement relative to normality and a 0 to 100-type response concept. However, the NHS asks specifically how normal the hip is today, and explicitly directs patients to consider its look, feel, and function. The NHS therefore seeks to measure perceived hip normality, whereas the SHV is framed as a subjective valuation of the hip. This distinction is subtle but clinically relevant, as the two questions may lead patients to consider different aspects of their recovery. In addition, the present study evaluates the NHS in an older hip-fracture population and examines its associations with established hip-specific and generic PROMs. The SHV provides useful conceptual precedent for a brief global hip outcome measure, but it does not remove the need to establish the construct validity, reliability, responsiveness, and interpretability of the NHS in the hip-fracture population.

The strong relationship between the NHS and OHS is particularly important because both measures capture aspects of hip-specific recovery, but they do so in different ways. The OHS assesses pain and functional limitation across 12 specific questions, whereas the NHS asks patients to make a single global judgement about how normal their hip feels and functions, considering look, feel, and function. The significant associations between the NHS and every individual OHS item suggest that this overall perception of normality reflects multiple aspects of hip recovery rather than being driven by a single symptom or functional limitation. The strongest relationships with standing, pain, night pain, and limping indicate that these readily identifiable aspects of hip function contribute substantially to patients’ overall perception of how normal their hip has become. The relatively weaker association with sudden severe pain may reflect the episodic nature of this symptom and its potentially lower influence on an overall assessment of hip normality. The presence of significant associations across all 12 OHS items provides supporting evidence that the NHS captures a meaningful aspect of hip-specific recovery rather than reflecting only a single domain of the OHS. The progressive increase in NHS across OHS quartiles provides further evidence that the NHS discriminates between patients with different levels of hip-related function. Therefore, the NHS should not be viewed simply as a shorter version of the OHS. Rather, the two measures address related but different questions. The OHS provides a multidimensional assessment of hip symptoms and function, whereas the NHS provides a global assessment of the patient’s perceived return to normality. Whilst the multidimensional assessment captured in the OHS provides a more specific investigation into the patient’s perception of specific hip outcomes, which is useful for furthering clinical understanding of outcomes following surgical management, this is of lesser value to a patient than a general return to health, as has been evidenced in other speciality fields [27]. When recognising this, in conjunction with previously documented work looking at patient perceptions of the OHS [28], the generalised global assessment which appears to be delivered by the NHS targets a specific outcome measure which has previously not been directly addressed.

The gradient in NHSs across OHS quartiles was consistent with the primary NHS–OHS correlation and therefore provides supportive rather than independent evidence of known-groups validity. Similarly, the item-level associations indicate that NHSs are related to multiple dimensions of hip symptoms and function, but they do not establish the independent contribution of any individual symptom or demonstrate that the NHS provides additional clinical information beyond established PROMs.

The relationship between the NHS and EQ-5D-5L further supports the construct validity of the measure while helping to define any potential role. The NHS was moderately correlated with the EQ-5D-5L index, and significant associations were observed across all five EQ-5D-5L dimensions. Mobility demonstrated the strongest relationship, followed by usual activities and self-care, which is consistent with the importance of mobility and independence in recovery following hip fracture [29]. Pain/discomfort and anxiety/depression showed weaker associations with NHSs, suggesting that perceived hip normality is influenced more strongly by physical function than by broader psychological or general health domains. Although the EQ-5D-5L index was significantly associated with NHS in univariable analysis, it was not independently associated with NHS after adjustment for OHS, age, sex, and time from fracture. In contrast, the OHS remained independently associated with NHS. This suggests that the relationship between overall health status and perceived hip normality is substantially accounted for by hip-specific symptoms and function. The NHS therefore appears to occupy a position closer to hip-specific recovery than to generic HRQoL, while still reflecting aspects of broader health status, providing support for the construct validity of the NHS as a measure of perceived hip normality. The multivariable findings should also be interpreted considering the strong correlation between OHS and EQ-5D-5L. The loss of an independent EQ-5D-5L association after adjustment for OHS may therefore reflect substantial overlap between the constructs represented by these measures and should not be interpreted as evidence that general health status is unrelated to perceived hip normality.

A potential advantage of the NHS is that it addresses a particular challenge in outcome assessment in patients following an acute hip fracture. The OHS and many other PROMs are commonly interpreted using change from a preoperative or pre-injury baseline. This approach is appropriate in elective hip surgery, where patients can complete a PROM before their planned operation and again following surgery. In hip fracture, however, the injury occurs unexpectedly, and patients rarely have a pre-fracture PROM available; the validity of this is debated. Although longitudinal change remains important when evaluating recovery, PROM guidance has also emphasised the value of considering the absolute level of pain, function and health status at follow-up rather than relying on change scores [10]. The NHS offers a different approach by asking patients directly how normal they consider their hip at the time of assessment. In this context, the NHS may have a role as a postoperative outcome measure that does not require a pre-fracture baseline. The single-item format is theoretically less burdensome than a 12-item questionnaire, but questionnaire completion time, acceptability and respondent burden were not measured in this study. The NHS may warrant investigation as a complementary global measure alongside established PROMs. Whether it can provide sufficient information for use without a multidimensional PROM remains an unanswered question requiring further study.

This study has several strengths. It evaluated the NHS in a clinically relevant hip-fracture population rather than an elective arthroplasty cohort and examined its relationship with both a hip-specific PROM and a widely accepted generic measure of HRQoL. The analysis extended beyond total scores to examine individual OHS items and EQ-5D-5L dimensions, providing insight into the components of recovery most closely associated with perceived hip normality. The use of known-groups analysis and multivariable regression provided additional evidence beyond simple correlation. The involvement of patients in the development and refinement of the NHS question and response format is an additional strength, supporting its patient-centred design.

However, several limitations should be acknowledged. This was a single-centre study with a relatively small sample, and the cross-sectional design limits the findings regarding responsiveness to recovery over time. The response rate was relatively low, with 85 of 196 eligible patients returning questionnaires (43.4%). This introduces potential response bias, particularly if patients who were frailer, cognitively impaired, more symptomatic, or otherwise less able to participate were less likely to provide PROM data. Patients unable to complete PROMs meaningfully were also excluded, further limiting generalisability to the full hip-fracture population. Where questionnaire assistance was provided, the potential for response characteristics to differ from those of independently completing patients must also be considered. Consequently, the present findings should not be assumed to apply equally to all patients following hip fracture. The timing of assessment varied from approximately 2 to 7 months following surgery, although there was no significant association between time from fracture and NHS. The absence of statistically significant associations between NHS and age, sex, or time from fracture should not be interpreted as evidence of generalisability across demographic groups or stability of NHSs over time. The assessment interval was relatively narrow (approximately 2–7 months), and the cross-sectional design does not permit conclusions regarding longitudinal stability or responsiveness. The distribution of NHSs showed limited floor effects, although 18.5% of scores were ≥90, indicating some concentration towards the upper end of the scale. Whether this represents a clinically important ceiling effect cannot be determined from the present cross-sectional data, and longitudinal studies are required to establish whether the scale can adequately detect further improvement. Several important psychometric properties of the NHS remain to be established, including test–retest reliability and the minimal clinically important difference (MCID). Although reliability of the SANE has not been specifically investigated in patients with hip pain, excellent test–retest reliability has been demonstrated in patients with shoulder pain [30]. Winterstein et al. [31] reported MCID values for the SANE that ranged from 7 to 19 at six- and 12-month follow-up, respectively, in patients with knee injuries. While these findings provide useful context, they cannot be directly extrapolated to the NHS or to an older hip-fracture population. Finally, the item-level analyses involved multiple statistical comparisons and should therefore be interpreted according to the magnitude and consistency of associations rather than statistical significance alone.

In conclusion, this study provides preliminary evidence of cross-sectional construct validity for the NHS following hip fracture surgery. NHSs were moderately associated with the OHS and EQ-5D-5L index, and exploratory analyses demonstrated associations across multiple domains of hip-related symptoms, function and general health status. These findings support further investigation of the NHS as a global measure of perceived hip normality, but do not establish responsiveness, test–retest reliability, clinically meaningful change, or suitability for standalone clinical use. Larger multicentre longitudinal studies with formal assessment of content validity, reliability, responsiveness and interpretability are required before the NHS can be considered a fully validated outcome measure.

## Figures and Tables

**Table 1:** Correlations between the Normal Hip Score and individual Oxford Hip Score items. Pearson correlation coefficients (r) between each of the 12 OHS items and the NHS in participants with paired NHS and OHS item data (n = 80). Higher OHS item scores indicate better hip-related health. Items are labelled according to the standard Oxford Hip Score questionnaire. Analysis population: n = 80 with complete NHS and OHS item data.

OHS Item	Pearson r with NHS	95% CI	*p* Value
1. Pain	0.586	0.420–0.714	<0.001
2. Washing/bathing	0.532	0.354–0.673	<0.001
3. Getting in/out of car	0.506	0.322–0.653	<0.001
4. Putting on socks/stockings	0.505	0.321–0.652	<0.001
5. Shopping	0.403	0.201–0.572	0.0002
6. Walking duration before severe pain	0.476	0.286–0.630	<0.001
7. Stairs	0.510	0.327–0.656	<0.001
8. Pain when rising from a chair	0.602	0.441–0.726	<0.001
9. Limping	0.559	0.387–0.694	<0.001
10. Sudden severe pain	0.294	0.079–0.483	0.0081
11. Work/housework interference	0.399	0.197–0.569	<0.001
12. Night pain	0.580	0.413–0.709	<0.001

All analyses, n = 80.

**Table 2:** Correlations between the Normal Hip Score and Oxford Hip Score, EQ-5D-5L index, and individual questionnaire dimensions. Associations between NHS and the total OHS, individual OHS items, EQ-5D-5L index, and individual EQ-5D-5L dimensions. Pearson correlation coefficients are reported for continuous measures and Spearman rank correlation coefficients where appropriate. Positive correlations indicate that higher scores on the comparator measure are associated with higher perceived hip normality; for EQ-5D-5L dimensions, negative correlations reflect greater impairment being associated with lower NHSs. *p* values are two-sided.

EQ-5D-5L Dimension	Pearson r	Spearman ρ	*p* Value
Mobility	−0.616	−0.624	<0.001
Self-care	−0.424	−0.415	<0.001
Usual activities	−0.510	−0.523	<0.001
Pain/discomfort	−0.341	−0.339	0.002
Anxiety/depression	−0.344	−0.321	0.004

**Table 3:** Multivariable linear regression analysis of factors associated with Normal Hip Score. Multivariable linear regression examining the adjusted associations of OHS, EQ-5D-5L index, age, sex, and time from fracture to assessment with NHSs. Results are presented as regression coefficients (β), 95% confidence intervals (CI), and *p*-values. The analysis included participants with complete data for all model variables (n = 79).

Predictor	β	95% CI	*p* Value
**Oxford Hip Score**	1.54	0.80 to 2.28	<0.001
**Age**	0.08	−0.36 to 0.53	0.720
**Sex**	−1.91	−11.36 to 7.53	0.688
**Time since fracture**	0.35	−5.84 to 6.54	0.911
**EQ-5D-5L index**	1.00	−24.74 to 26.73	0.939

## Data Availability

Data is available on reasonable request to corresponding author due to data governance requirements.
